# Mitigating Radiation Damage to Polyethylene in Transmission Electron Microscopy by Free Radical Scavengers

**DOI:** 10.1021/acsomega.5c09428

**Published:** 2025-11-24

**Authors:** Hsiao-Fang Wang, Yen-Chi Ho, Yi-Cen Shih

**Affiliations:** Department of Chemical and Materials Engineering, 34911 National Central University, No. 300, Zhongda Rd., Zhongli District, Taoyuan City 320317, Taiwan, ROC

## Abstract

Polyethylene (PE) is the most extensively used semicrystalline polymer due to its wide array of applications. The properties of PE are influenced by its nanosized crystals and amorphous regions. Transmission electron microscopy (TEM) is a vital tool for revealing the nanostructures within materials. However, high-energy incident electrons can inflict radiolysis damage through electron irradiation, resulting in the loss of crucial information regarding crystallization. In this study, we examine the effect of free radical scavengers on PE as an approach to mitigating beam damage. Quantifying the beam damage by critical dose (*D*_c_) values from the decay of electron diffraction (ED) peaks shows that the beam damage of PE in TEM can be mitigated by casting an aqueous solution containing a free radical scavenger on PE crystals. The protective effect of the free radical scavenger is attributed to the efficient scavenging of reactive radical species by its hydroxyl group on phenols. This study highlights a nonincorporative approach into materials to mitigate the beam damage by the coating of free radical scavengers on all kinds of materials.

## Graphical abstract

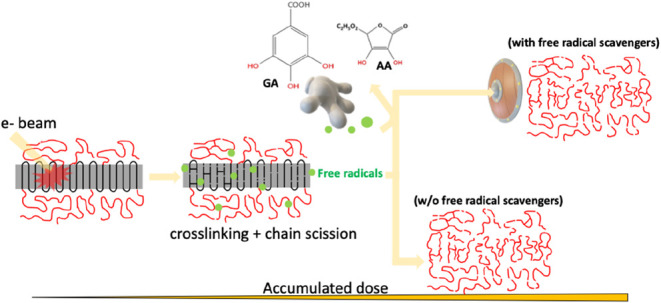

## 1 Introduction

Semicrystalline polyethylene (PE) is an essential thermoplastic polymer that stands out for its remarkable chemical resistance, outstanding flexibility, and lightweight characteristics. Understanding PE’s crystallinity and type of crystal is essential not only for materials scientists but also for industries that rely on its diverse properties.^1,2^

Transmission electron microscopy (TEM) is an advanced analytical technique utilized to visualize and characterize materials through imaging, diffraction, and spectroscopy. However, soft matter, such as polymers, is sensitive to the high-energy electron beam. The appearance of the specimen changes considerably during observation in TEM because of radiation damage. Radiation damage can be categorized as elastic scattering and inelastic scattering. Elastic scattering can result in electrostatic charging, atomic displacement, and e-beam sputtering. Inelastic scattering can produce radiolysis effects (structural damage or mass loss), specimen heating, and hydrocarbon contamination.^3–5^ In soft matter, the primary type of radiation damage is radiolysis, which results in the formation of free radicals. Subsequently, chemical bonds are broken or cross-linked, causing the loss of crystallinity or image contrast. The effects of radiolysis greatly limit the ability to conduct in situ observations to examine the dynamic behaviors of materials.

Cryogenic conditions can effectively minimize radiation damage in TEM by limiting the diffusion of free radicals.^5,6^ However, the experiments requiring temperature as a key parameter cannot be conducted at cryogenic temperatures, such as order–order transition of the block copolymer,^7^ solid–solid phase transformations in organic semiconductors,^8^ and liquid cell.^9,10^ Technically, changing the accelerating voltage and lowering the dose rate are the ways to reduce electron irradiation damage.^4,11,12^ For organic specimens, utilizing a higher accelerating voltage combined with a low dose is an effective approach to suppressing radiation damage. However, this method results in a lower contrast due to the reduction of the cross-section for elastic scattering.

Antioxidants, also known as free radical scavengers, are substances that effectively neutralize free radicals, protecting materials from radiation damage. They have been used to reduce the radiation damage. For example, graphene and isopropanol as free radical scavengers have been utilized in liquid cell environments to neutralize radicals generated by the radiolysis of water molecules during the TEM experiment.^13,14^ Blends of free radical scavengers have also been used to prevent irradiation damage during γ-irradiation and electron beam irradiation. Phenolic free radical scavenger blends in PE demonstrate that the radiation yield of cross-linking can be reduced.^15^ Blending free radical scavengers into conjugated polymers can help minimize beam damage.^16^ However, the presence of liquid environments restricts the options for investigating the solid state. Blends of free radical scavengers may cause changes in the intrinsic properties of materials.

Here, we propose that free radical scavengers can be used to reduce the beam damage of PE in TEM at room temperature. We chose gallic acid (GA) and ascorbic acid (AA) as free radical scavengers because the hydroxyl group on phenols has been shown to react with free radicals.^17–19^ We adopt electron diffraction (ED) to reveal the damage to the crystalline structure under the accumulated dose. By applying a thin layer of the aqueous solution containing a free radical scavenger on the PE crystal, we demonstrate that the critical dose (*D*_c_) for damage increases with the addition of these additives, indicating a reduction in radiation damage, even when the free radical scavengers do not incorporate into the crystals.

## 2 Materials and Methods

### 2.1 Materials

The PE sample was provided by LCY Chemical Corp. The number-average molecular weight (*M_n_*) was 48,700 g/mol, and *M*_*w*_/*M*_*n*_ was 1.37. GA (purtily >99.9%) and AA (purtily >99.9%) were purchased from Sigma-Aldrich.

### 2.2 Sample Preparation

The PE was dissolved in boiling (180 °C) decalin (99%, Sigma-Aldrich) at 0.5 wt %. The PE thin films were prepared by spin-coating (1000 rpm, 60 s) on a freshly cleaved mica substrate. After evaporation of decalin, the PE thin films were annealed at 150 °C for 2 h to crystallize. The formation of spherulites was found using polarized optical microscopy and TEM (Figure S1). The PE thin films/mica were gently immersed in water. The PE thin films were exfoliated from the mica substrate and floated on the surface of the water. The PE thin films were collected on a copper grid (100 mesh) with a carbon-supporting film. Water-soluble GA and AA were dissolved in distilled water at 1.6 wt %. A plasma treatment (PECLO, easiGlow) was performed to enhance the hydrophilicity of the PE films. Subsequently, 0.15 mL of an aqueous solution of GA or AA was dropped onto the PE film to form a thin layer. Samples were dried overnight at room temperature (25 °C) under a vacuum. We note that the aqueous solution was selected as a nondestructive approach for polymers, preserving the polymer’s intrinsic properties.

### 2.3 Electron Diffraction (ED)

ED measurements were performed using a JEM-2100 (JEOL Ltd.) at an acceleration voltage of 200 kV with a US1000 camera (2k × 2k) (Gatan, Inc.). ED patterns were recorded with the dose rate ∼1.0 e^–^/Å^2^·s. The exposure time per pattern was 0.5 s, and the acquisition interval was 3.0 s. The selective area aperture was ∼1.02 μm^2^.

The ED patterns were azimuthally averaged to obtain the diffraction intensity curves and model the curves using least-squares fitting.^20^ By subtracting a power-law background from the azimuthal-averaged intensity profile, the remaining diffraction signals can be accurately modeled using three Gaussian peaks (*R* ^2^ > 99.5%). These peaks correspond to the amorphous structure and the 110 and 200 diffraction peaks. The total diffraction intensity can be expressed as follows

1 $$I=A_{a}e^{-(x-{b_{a})}^{2}/{c_{a}}^{2}}+A_{110}e^{-(x-{b_{110})}^{2}/{c_{110}}^{2}}+A_{200}e^{-(x-{b_{200})}^{2}/{c_{200}}^{2}}+dx^{-\alpha }=I_{\mathrm{amor}\mathrm{phous}}+I_{\mathrm{crystal}}$$

where *A* is the amplitude of the Gaussian curve, *a* is amorphous, *b* is the Gaussian peak position, *c* is the width of the Gaussian curve, and α is related to the power-law. The total crystal diffraction intensity is *I*_crystal_ = *I*_110_ + *I*_200,_and the minor 020 diffraction peaks are not considered because 020 becomes invisible at low accumulated doses. The relative intensities of *I*_crystal_ and *I*_amorphous_ are plotted from the area of the fitted Gaussian peaks on the azimuthally averaged diffraction patterns, normalized by the maximum intensity of the whole series.

## 3 Results and Discussion

Typical ED patterns taken from a neat PE film at 298 K are presented in Figure 1A. The pristine film (0.5 e/Å^2^ dose for acquiring the first ED pattern) shows a polycrystalline PE structure with sharp arcs corresponding to 110, 200, and 020 diffraction. The appearance of diffraction arcs instead of complete rings indicates the preferential orientation of the crystal. The intensities of the 110, 200, and 020 diffractions decrease with increasing dose from 0.5 to 42.1 e^–^/Å^2^. At 42.1 e^–^/Å^2^, the pattern becomes a halo pattern in which 110 and 200 diffractions are indistinguishable. Figure 1B shows the azimuthally averaged ED profile with the accumulated doses. The background-subtracted intensities of the 110 and 200 peaks decrease with an increasing irradiation dose. In addition, 110, 200, and amorphous peaks shift toward larger spacings (lower *q* values) with accumulated doses (Figure 1C). The 110 diffraction peak shifts linearly from 2.21 to 2.09 nm^–1^ after accumulating 34.5 e^–^/Å^2^, indicating the lattice expansion of ∼0.6%. The 200 diffraction peak drops dramatically from 2.43 to 2.22 nm^–1^, corresponding to the lattice expansion of ∼0.9%. The amorphous peak shows a relatively small expansion (∼0.4%), suggesting that the amorphous region undergoes mild beam damage. These results reveal that the crystalline regularity decreases with electron irradiation, suggesting a change in crystalline structure due to chain scission and cross-linking.^21,22^ The cleavage of C–H bonds leads to lattice expansion due to the formation of unsaturated C=C bonds and the rotation of adjacent C–H bonds. This process results in an increased distance between the two hydrogen atoms. As the number of C=C bonds increases, more hydrogen atoms protrude, pushing neighboring PE chains apart and resulting in enlarged lattice spacing.^23–26^ Cross-linking between molecular chains in the PE crystal results in the local expansion of the crystal lattice due to the increasing lateral distance of adjacent chains or emergence of defects in crystalline regions.^27,28^ A combination of chain scission and cross-linking leads to lattice expansion due to electron beam damage.

**Figure 1:**
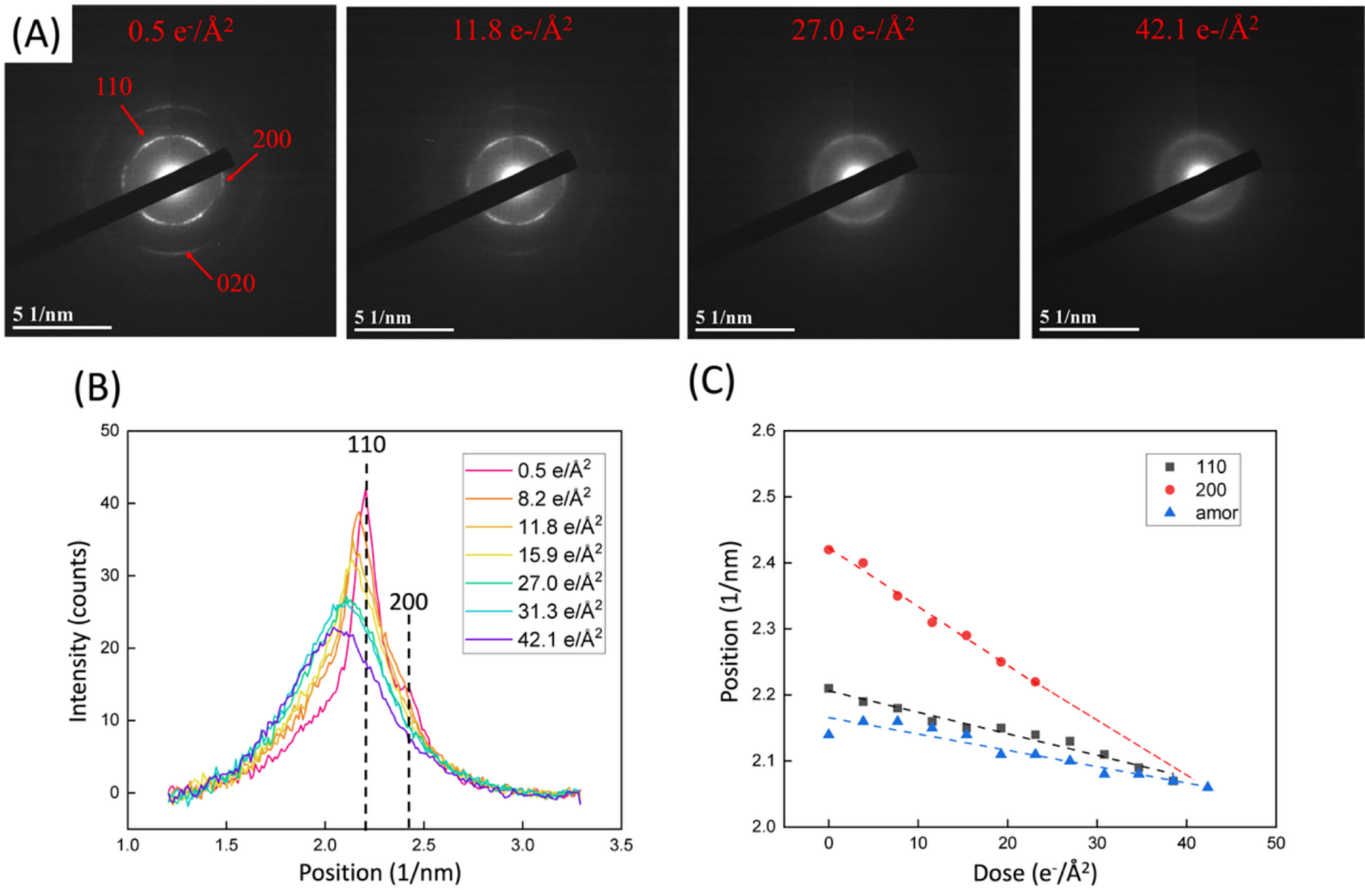
(A) ED patterns of a PE sample with dose rate = 1.0 e^–^/Å^2^·s from 0.5 to 42.1 e^–^/Å^2^. (B) Azimuthal-averaged intensity profiles of ED patterns from PE with accumulated electron doses. (C) Peak positions of PE 110 (black square), 200 (red circle), and amorphous (blue triangle) peaks with increasing accumulated electron doses.

Mitigation of beam damage was characterized in PE crystals with and without free radical scavengers. The chemical structures of PE, GA, and AA (free radical scavengers) are shown in Figure 2A. By addition of free radical scavengers through drop coating, as shown in Figure 2B–D, 110 and 200 peaks can be found in ED patterns, indicating that this nonincorporative approach does not change the crystal structure. The 110 and 200 peaks for neat PE and PE + AA fade away as the dose accumulates to 30.7 e^–^/Å^2^, indicating the loss of the detected crystal signal. Two peaks were partially preserved for PE + GA, indicating that some crystal structure remained intact to some extent. The GA and AA diffractions can be found in ED patterns (Figure 2C,D) and azimuthal-averaged intensity profiles (Figure S2). TEM and AFM images further indicated the existence of GA or AA on the PE crystal (Figure S3). Figure 2E,F shows the intensity of *I*_crystal_ and *I*_amorphous_ from PE as a function of accumulated doses. The *I*_crystal_ revealed an exponential decay of the diffraction intensity, whereas the *I*_amorphous_ exhibited an exponential increase of intensity through amorphization of the crystal.^4,29^ To quantitatively characterize beam damage in PE, PE + GA, and PE + AA, we calculated the critical dose (*D*_c_) values, defined as the dose at which the relative diffraction intensity drops to 1/*e* (∼37%) of its initial value, to assess the decay of ED intensities. *D*_c_ values are 24.6, 30.9, and 26.7 e^–^/Å^2^ for neat PE, PE + GA, and PE + AA, respectively (Figure 2F). The addition of GA and AA increases *D*_c_ by factors of 1.26 and 1.09, respectively. The results show that GA and AA mitigate the beam damage by quenching the free radicals generated by exposure to the electron beam (see Section 4 for details).

**Figure 2:**
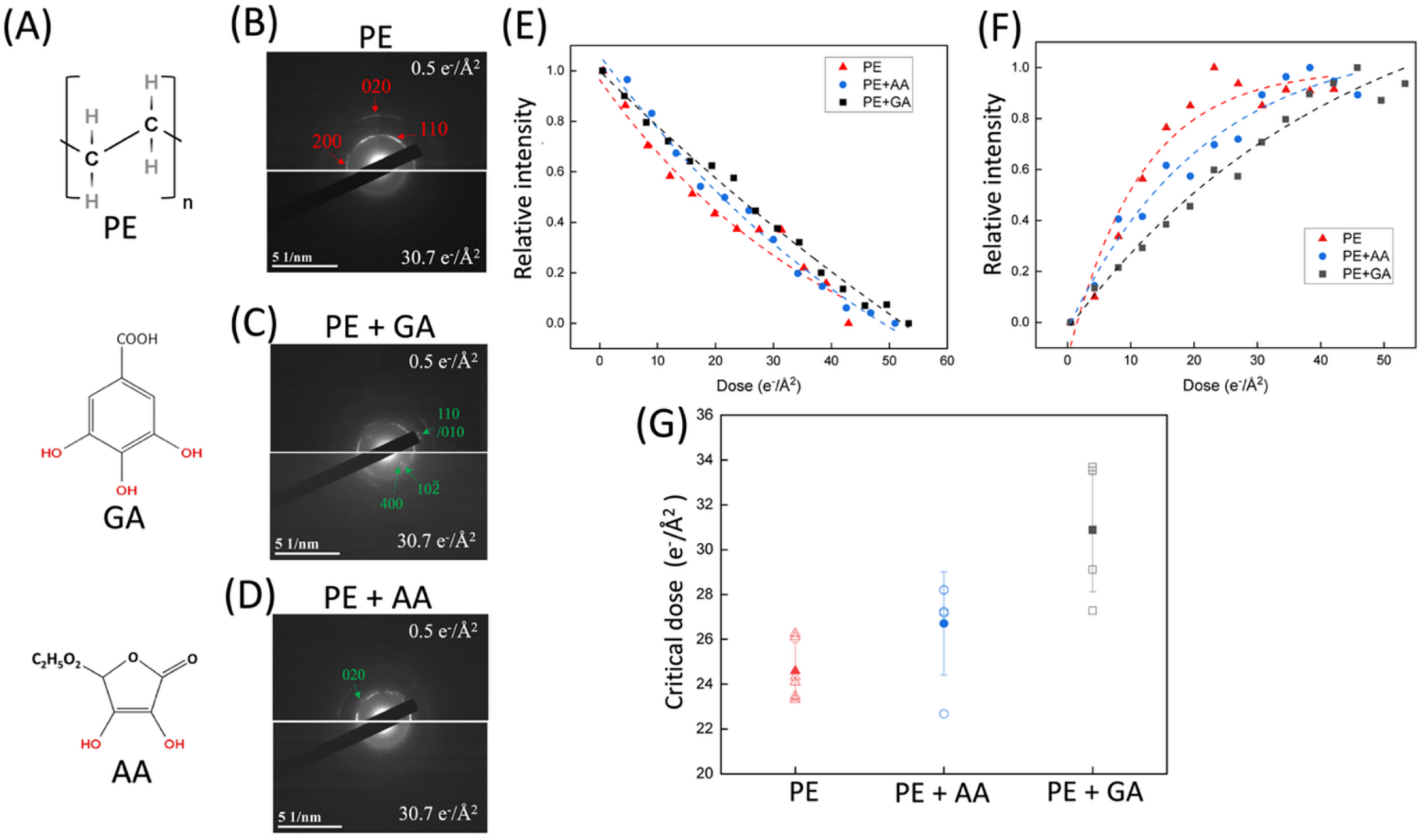
(A) Chemical structures of the PE polymer and free radical scavengers used in this study. The red hydroxyl groups on the phenols represent the sites that can react with free radicals. ED patterns of (B) neat PE, (C) PE + GA, and (D) PE + AA at low (0.5 e^–^/Å^2^) and high dose (30.7 e^–^/Å^2^), showing loss of observed crystal signal for neat PE and PE + AA and partially preserved crystal structure for PE + GA. Red arrows indicate the diffractions of PE. Green arrows indicate the diffractions for GA and AA. Relative intensity of (E) *I*_crystal_ and (F) *I*_amorphous_ as functions of accumulated dose for PE (red triangle), PE + GA (black square), and PE + AA (blue circle). (G) Critical doses (*D*_c_) of PE, PE + GA, and PE + AA.

In the late stages of beam exposure, the ED patterns distinctly transformed into an amorphous halo, demonstrating an amorphization of the PE crystal (Figures S4–S6). The amorphization of the PE crystal shows the increase in *I*_amorphous_ with accumulated doses (Figure 2G). *I*_amorphous_ shows an exponential increase in the amorphous phase and saturated 27.0 and 30.7 e^–^/Å^2^ for PE and PE + AA, respectively, reflecting the process of PE amorphization. By contrast, at the upper-limited accumulated dose, *I*_amorphous_ of PE + GA does not exhibit the saturation point. The tendency of amorphization rate can be characterized by the amorphization rate constant exponential curve

2 $$I\left(D\right)=I_{0}\cdot e^{-k\left(D\right)}$$

3 $$\ln\left(I\left(D\right)\right)={\ln I}_{0}-\mathrm{kD}$$

where *D* is the accumulated dose, *I*_0_ is the initial intensity, and *k* is the amorphization rate constant. The *k* values are 0.032, 0.012, and 0.014 for neat PE, PE + GA, and PE + AA, respectively, suggesting the slower amorphization due to the addition of free radical scavengers. Furthermore, PE + GA provides greater protection against beam damage than PE + AA, as evidenced by the slower rate of amorphization.

## 4 Discussion

A high-energy electron beam generated the free radicals and diffused in the PE polymer, causing beam damage to the material. The addition of free radical scavengers quenches reactive free radicals and increases the lifetime of the PE crystal (Figure 3). Cross-linking and chain scission occur in PE when it is exposed to the electron beam, at which the *G* value can be used to quantify the chemical yield of cross-linking and chain scission resulting from the radiation. The *G* value is defined as the chemical yield of radiation in the number of molecules reacted per 100 eV of absorbed energy. The *G* values for cross-linking *G*(*X*) and chain scission *G*(*S*) for PE are 0.96–1.42 and 0.19–0.48, respectively.^21^ *G*(*S*):G­(*X*) < 1.0 indicates that PE is favored for cross-linking rather than chain scission.^30,31^

**Figure 3:**
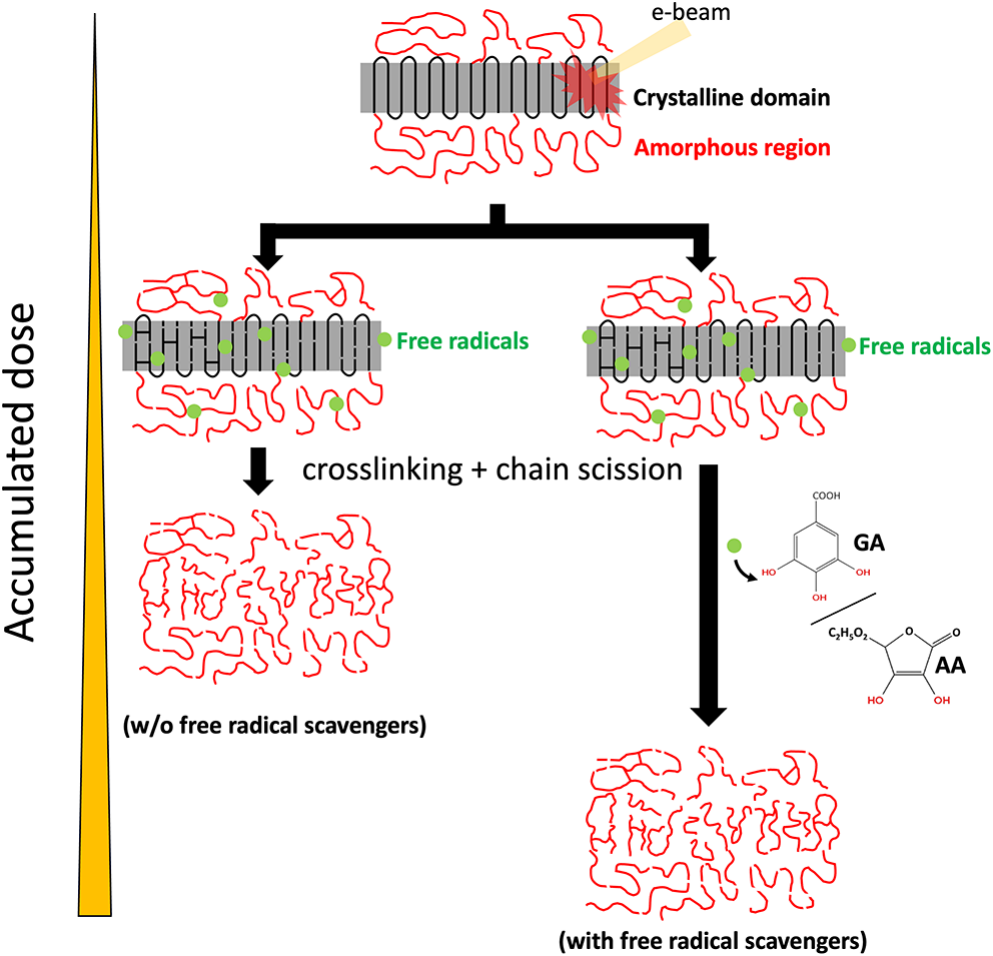
Schematic of beam damage in the PE crystal. Gray regions and red chains represent crystalline domains and amorphous domains, respectively. Exposure to the electron beam generates a number of free radicals and reacting species in the PE crystal, resulting in cross-linking and chain scission reactions. Without free radical scavengers, beam damage is severe. With free radical scavengers, GA and AA, free radicals can be quenched and further mitigate the beam damage.

Figure 4 shows the radiation effect on the PE, in which cross-linking and chain scission occur simultaneously. The initiation of (1) cross-linking involves the homolytic cleavage of the C–H bond of the PE backbone, generating macroradicals (PE^•^) and H^•^. When two such macroradicals are in proximity, they recombine to form a cross-linking network and hydrogen. At the same time, the generated radicals undergo random scission of the C–C and C–H bonds. We note that the number of radicals should be less than one for cross-linking due to the *G* value. (2) Homolytic cleavage of the C–C bond yields two PE^•^ with lower molecular weight (*M_n_*). Hydrogen abstraction between two PE^•^ form the PE with a C=C chain end and with a lower *M_n_*. (3) Above PE^•^ also attaches C–H in the adjacent PE backbone, resulting in the other PE^•^ and PE with a lower *M_n_*. (4) C–H bond scission forms the H^•^ and secondary PE radicals that undergo β-scission. These secondary PE radicals form PEs with C=C either in the middle of the chain or at the chain end.

**Figure 4:**
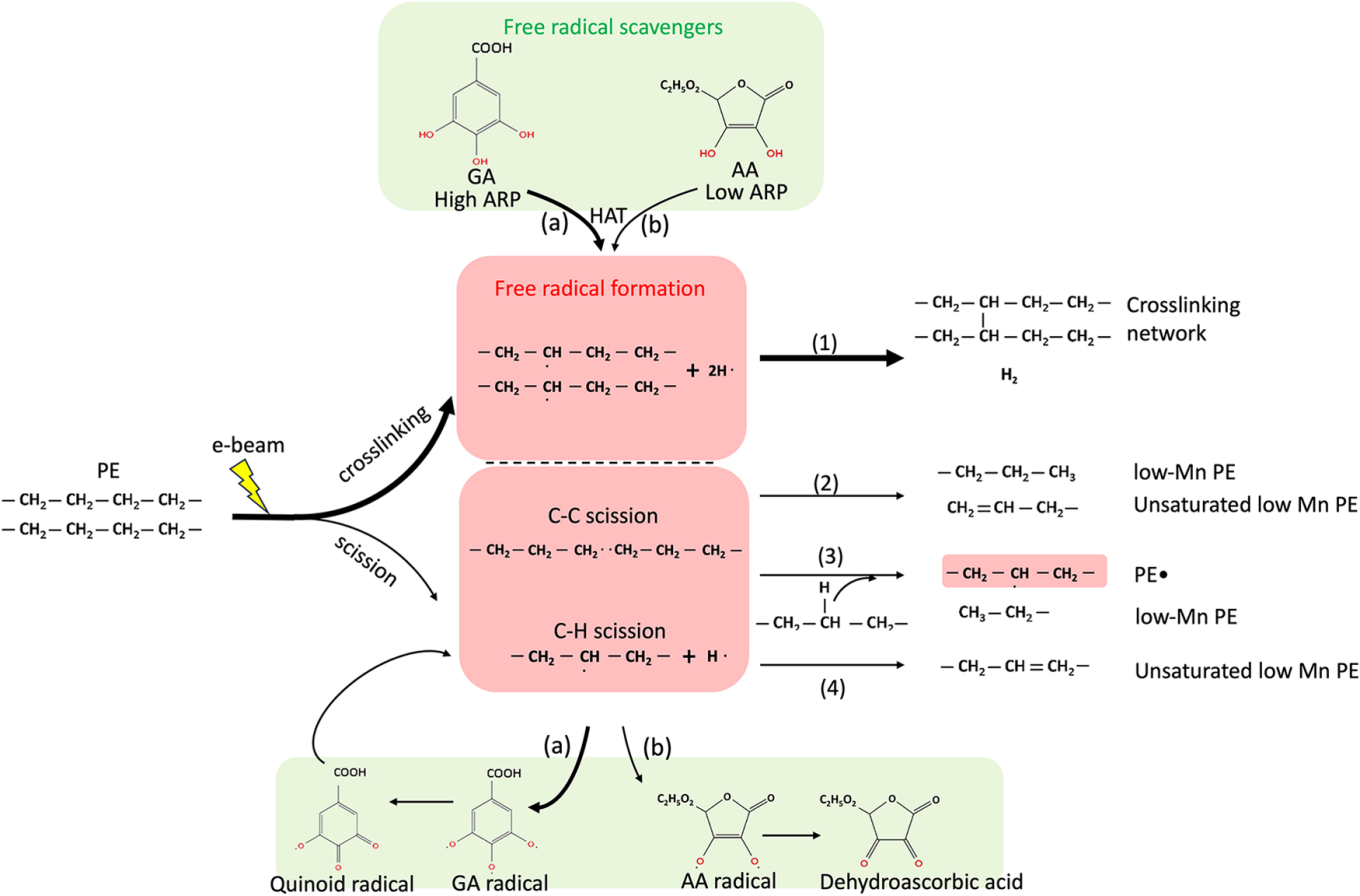
Proposed mechanisms for cross-linking and chain scission reactions in PE during exposure to electron beam (pathways (1), (2), (3), and (4)), and how (a) GA and (b) AA quench free radicals through the hydrogen atom transfer (HAT) mechanism.

As shown in the red rectangle of Figure 4, PE generates different types of free radicals due to the high-energy electron beam. The addition of free radical scavengers quenches free radicals before they cause further damage to the PE crystal (green rectangle in Figure 4). GA and AA use the hydrogen atom transfer (HAT) mechanism to react with free radicals, yielding a more stable radical or a nonradical species. GA is more effective at mitigating beam damage because of a higher number of hydroxyl groups on the aromatic ring. As illustrated in pathway (a), GA donates three hydrogen atoms from its phenolic hydroxyl group to quench reactive radicals from PE, generating a relatively stable gallic acid radical. This radical is stabilized through resonance delocalization over the aromatic ring system.^32^ After that, the GA radical forms the quinoid radicals and further reacts with the reactive radicals from PE.^33^ Pathway (b) indicates that AA donates two hydrogen atoms from its phenolic hydroxyl group to react with free radicals, forming a relatively stable ascorbate radical. This radical intermediate undergoes further delocalization to form dehydroascorbic acid.^34^

Figure 5 shows the plot of the difference in *D*_c_ (Δ*D*_c_) with and without free radical scavengers for the mitigated effect of beam damage, comparing the simple casting method used in this study with the polymer-small molecular blend approach described in the literature.^16^ Gomez et al. examine the *D*_c_ of conjugated polymers with and without free radical scavengers. 7 wt % free radical scavengers, butylated hydroxytoluene (BHT), and 2,2,6,6-tetramethyl-1-piperidinyloxy (TEMPO) were blended into conjugated polymers (poly­(3-hexylthiophene-2,5-diyl) (P3HT), poly­(3-dodecylthiophene-2,5-diyl) (P3DDT), and poly­[(5,6-difluoro-2,1,3-benzothiadiazol-4,7-diyl)-*alt*-(3,3″′-di­(2-octyldodecyl)-2,2′;5′,2″;5″,2″′-quaterthiophene-5,5″′-diyl)] (PffBT4T-2OD)). They speculated that the addition of free radical scavengers resides in amorphous regions and does not perturb the crystal structure. The Δ*D*_c_ values are 7.8, 5.3, 7.2, and 5.5 e^–^/Å^2^ for PffBT4T-2OD + BHT, PffBT4T-2OD + TEMPO, P3HT + BHT, and P3DDT + BHT, respectively. In this study, Δ*D*_c_ values are 6.3 and 2.1 e^–^/Å^2^ for PE + GA and PE + AA, respectively. Although the Δ*D*_c_ of the addition of GA is smaller than that of some of the blend samples, the quenching efficiency of GA for free radicals through simple casting is comparable to that of the blend approach (PffBT4T-2OD + TEMPO and P3DDT + BHT). Using the casting approach, we speculate that free radical scavengers reside on the surface of the stacked PE lamellar crystal as well as the amorphous region (Figure 5B), further protecting the PE crystal during irradiation.

**Figure 5:**
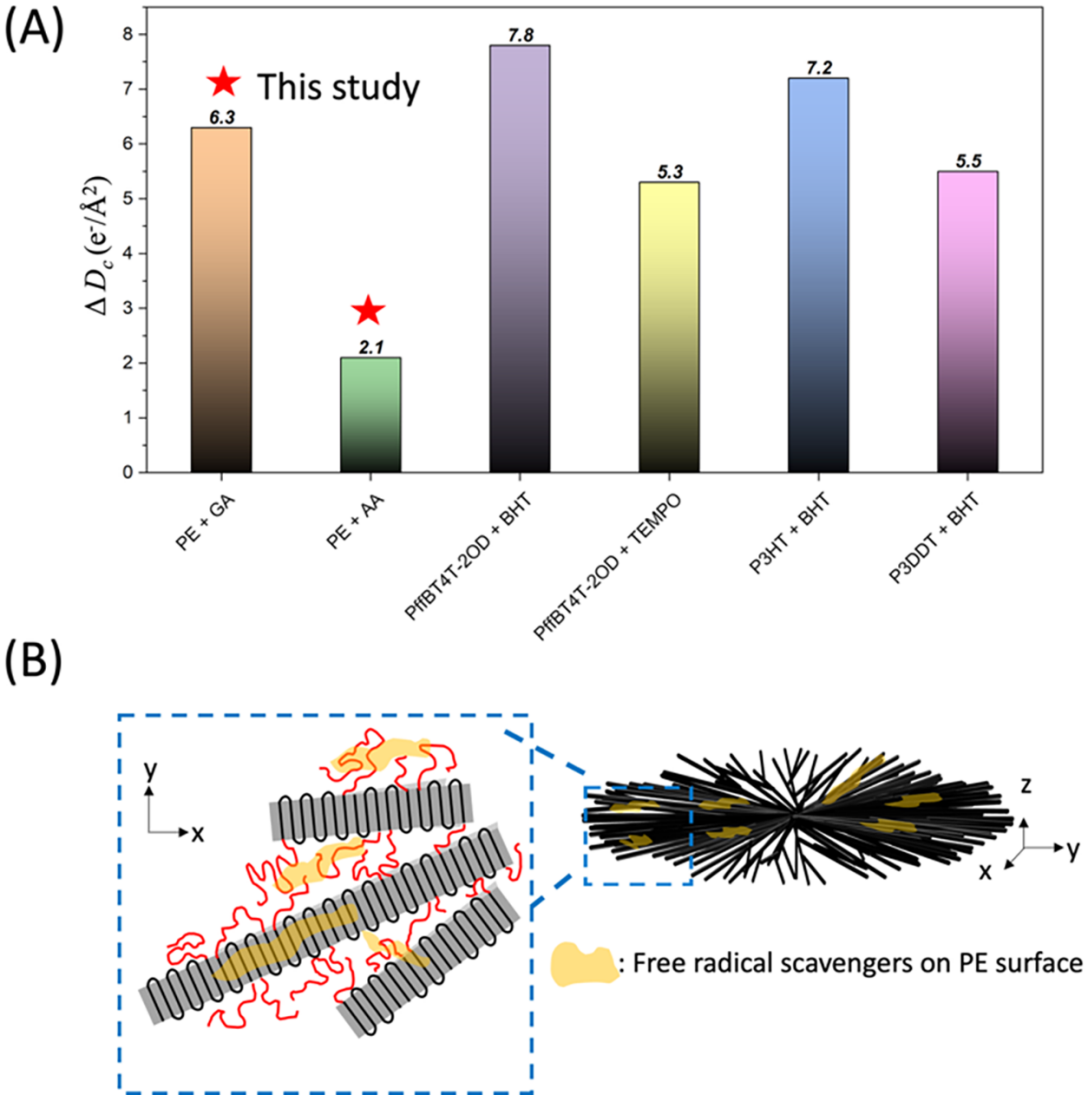
(A) Difference in critical doses (Δ*D*_c_) of PE + GA, PE + AA, PffBT4T-2OD + BHT, P3HT + BHT, and P3DDT + BHT with and without free radical scavengers. The value outside the bar displays the Δ*D*_c_ value. (B) Schematic of the distribution of free radical scavengers on PE spherulites. Gray regions and red chains represent crystalline domains and amorphous domains, respectively. The yellow regions represent the distribution of free radical scavengers on the surface of the stacked PE lamellar crystal and the amorphous region.

By contrast, the free radical scavengers only exist in the amorphous region in the blending approach.^16^ However, the area protected by free radical scavengers in this study cannot be controlled because GA and AA tend to form aggregates. This aggregation leads to a variation in the protective effect, as shown in Figure 2G. Moreover, although BHT has only one hydroxyl group, it shows a high *D*_c_ in PffBT4T-2OD + BHT and P3HT + BHT due to the high concentration of free radical scavengers. Increasing the concentration of free radical scavengers does not necessarily ensure an improvement in the *D*_c_.^16^ It is also possible that a high concentration of additions increases the film thickness, which constrains the imaging capability (Figure S7). It would be necessary to determine the optimal concentration in order to maximize the protective effect of the free radical scavenger.

Moreover, we noted that the blend approach may lead to cocrystallization or disruptions in the materials,^35–37^ leading to a loss of their intrinsic behavior. The present study demonstrated that a thin surface coating of GA or AA essentially stabilizes the polymer crystalline structure. An advantage of the present approach is that it highlights a nonincorporative strategy to mitigate electron beam damage, offering a surface-based alternative to conventional bulk stabilization methods.

## 5 Conclusions

In this study, we demonstrated that free radical scavengers can increase the critical doses of diffraction experiments. Water-soluble free radical scavengers can mitigate beam damage in semicrystalline polymers at room temperature through the nonincorporative approach between the polymer crystal and free radical scavengers. Free radical scavengers quench the free radicals of PE generated from the electron beam. A higher number of hydroxyl groups on the aromatic ring of free radical scavengers stabilized more free radicals. As a result, we can use free radical scavengers to conduct experiments requiring high-dose exposure without altering intrinsic properties. This research will facilitate the advancement of electron microscopy for observing beam-sensitive soft materials, allowing for an enhanced observation time and enabling time-resolved experiments.

## Supplementary Material



## Notes

Polarized optical microscopy image and TEM micrographs of PE spherulites (Figure S1); azimuthal-averaged intensity profiles of ED patterns at 0.5 e^–^/Å^2^ (dose rate = 1.0 e^–^/Å^2^·s) from PE + GA and PE + AA (Figure S2); TEM and AFM images of PE + GA and PE + AA (Figure S3); representative ED patterns from the PE spherulites, PE + GA, and PE + AA taken at increasing doses of irradiation (Figures S4–S6); polarized optical microscopy image and TEM micrographs of PE + GA at low (1.6 wt %) and high concentrations (10 wt %) (Figure S7); other data for the relative intensity of *I*_crystal_ as a function of accumulated dose for PE, PE + GA, and PE + AA (Figure S8) (PDF)
