# Correction to “Mitigating Radiation Damage to Polyethylene in Transmission Electron Microscopy by Free Radical Scavengers”

**DOI:** 10.1021/acsomega.5c12687

**Published:** 2025-12-27

**Authors:** Hsiao-Fang Wang, Yen-Chi Ho, Yi-Cen Shih

In section 2.1 (Materials), “by LCY Chemical Corp” should be “by Formosa Plastics Corporation (FPC)”.

In section 3 (Results and Discussion), second paragraph, “*D*_c_ values are 24.6, 30.9, and 26.7 e^–^/Å^2^ for neat PE, PE + GA, and PE + AA, respectively (Figure 2F)” should be “*D*_c_ values are 24.6, 30.9, and 26.7 e^–^/Å^2^ for neat PE, PE + GA, and PE + AA, respectively (Figure 2G).”

