# Supplementary material for: Mitigating Radiation Damage to Polyethylene in Transmission Electron Microscopy by Free Radical Scavengers
Source: ACS Omega. 2025 Nov 24;10(48):59685–91. doi: 10.1021/acsomega.5c09428 (PMC12771416; doi:10.1021/acsomega.5c09428)
Supplement: Supplementary file 1 [file ao5c09428_si_001.pdf]

Supplementary Information

Mitigating Radiation Damage to Polyethylene in Transmission Electron Microscopy by Free Radical Scavengers

Hsiao-Fang Wang[1,], Yen-Chi Ho[1] and Yi-Cen Shih[1]*

[1] Department of Chemical and Materials Engineering, National Central University, No. 300, Zhongda Rd., Zhongli District, Taoyuan City 320317, Taiwan (R.O.C.)

[*] Corresponding author. E-mail address: hsiaofangwang@ncu.edu.tw

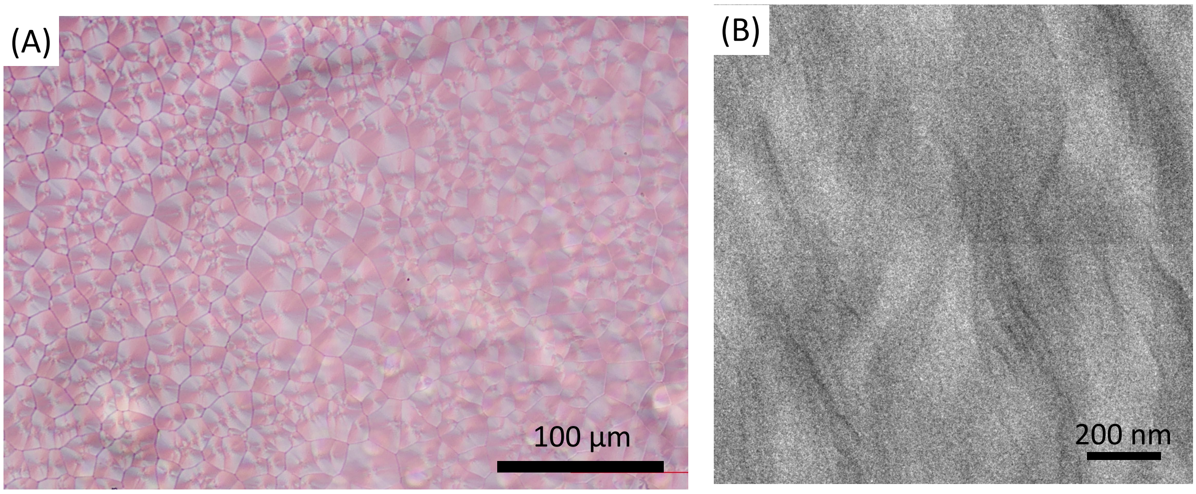

Figure S1. (A) Polarized optical microscopy image and (B) TEM micrograph of PE spherulites.

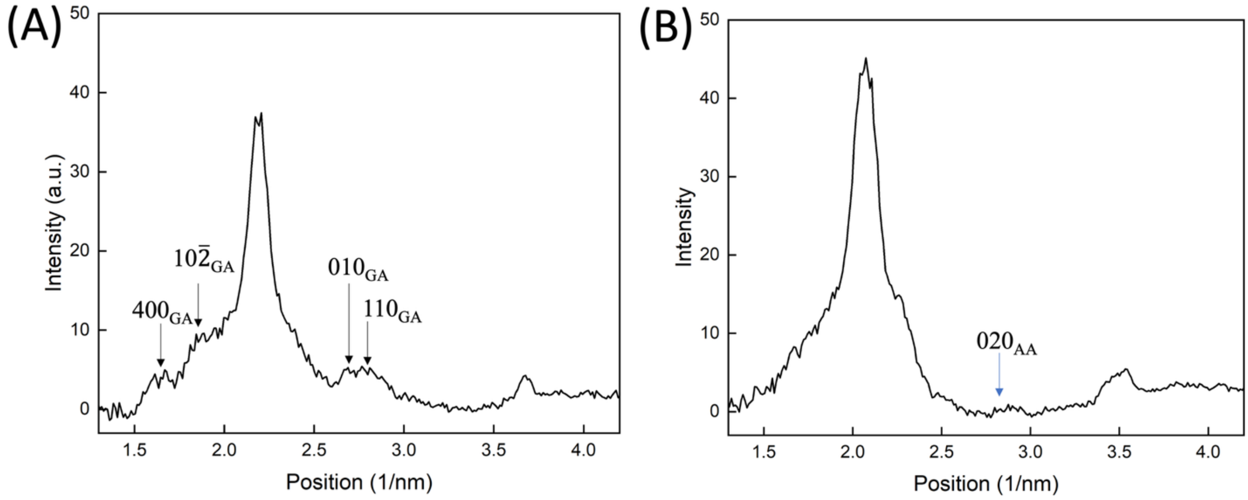

Figure S2. Azimuthal-averaged intensity profiles of ED patterns at 0.5 $e^-/Å^2$ (dose rate = 1.0 $e^-/Å^2 \cdot sec$) from (A) PE + GA and (B) PE + AA. The arrows indicate the diffraction peaks for GA (left figure) and AA (right figure).

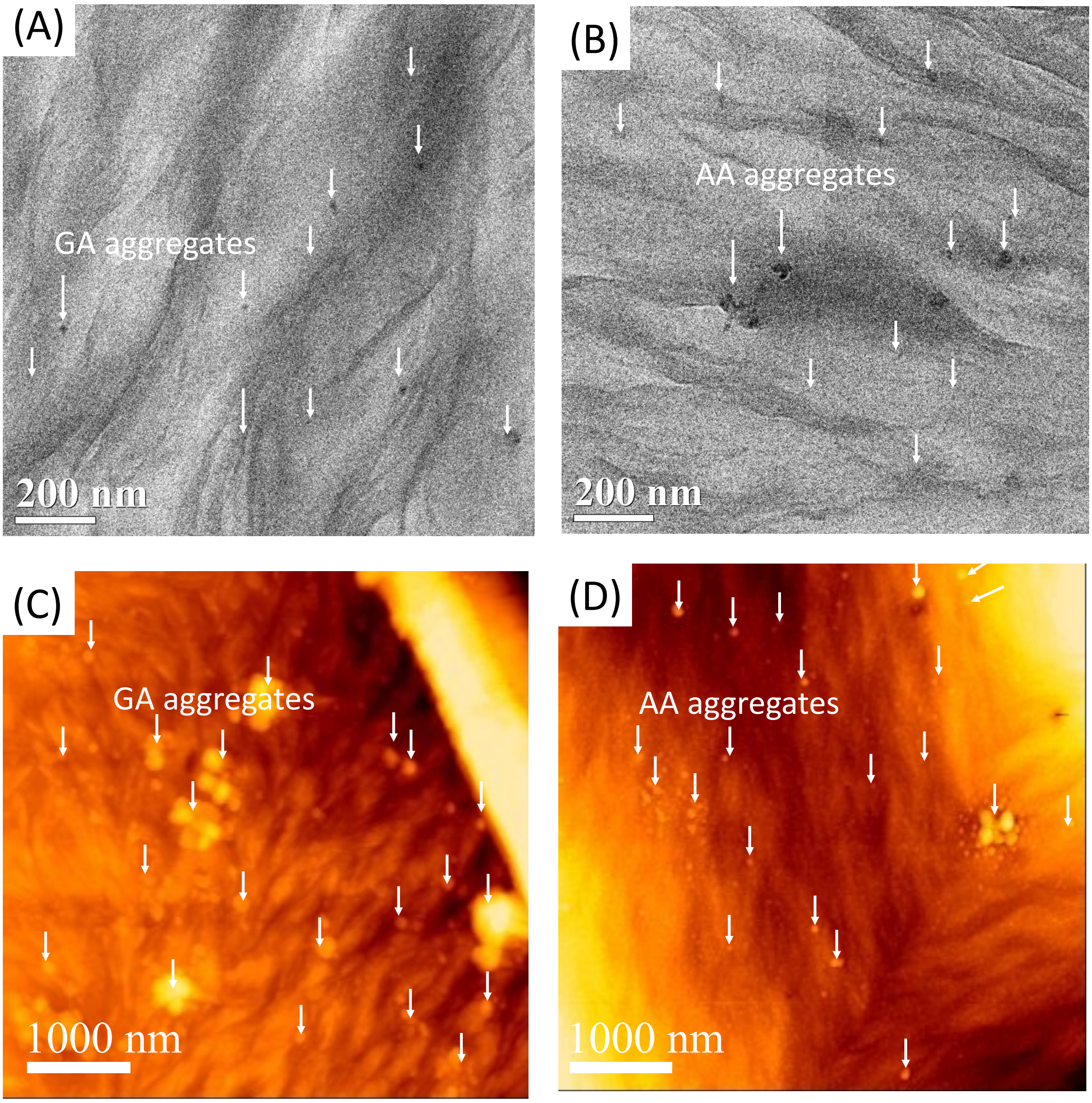

Figure S3. TEM and AFM images of (A, C) PE + GA and (B, D) PE + AA. The white arrows indicate the GA and AA aggregates.

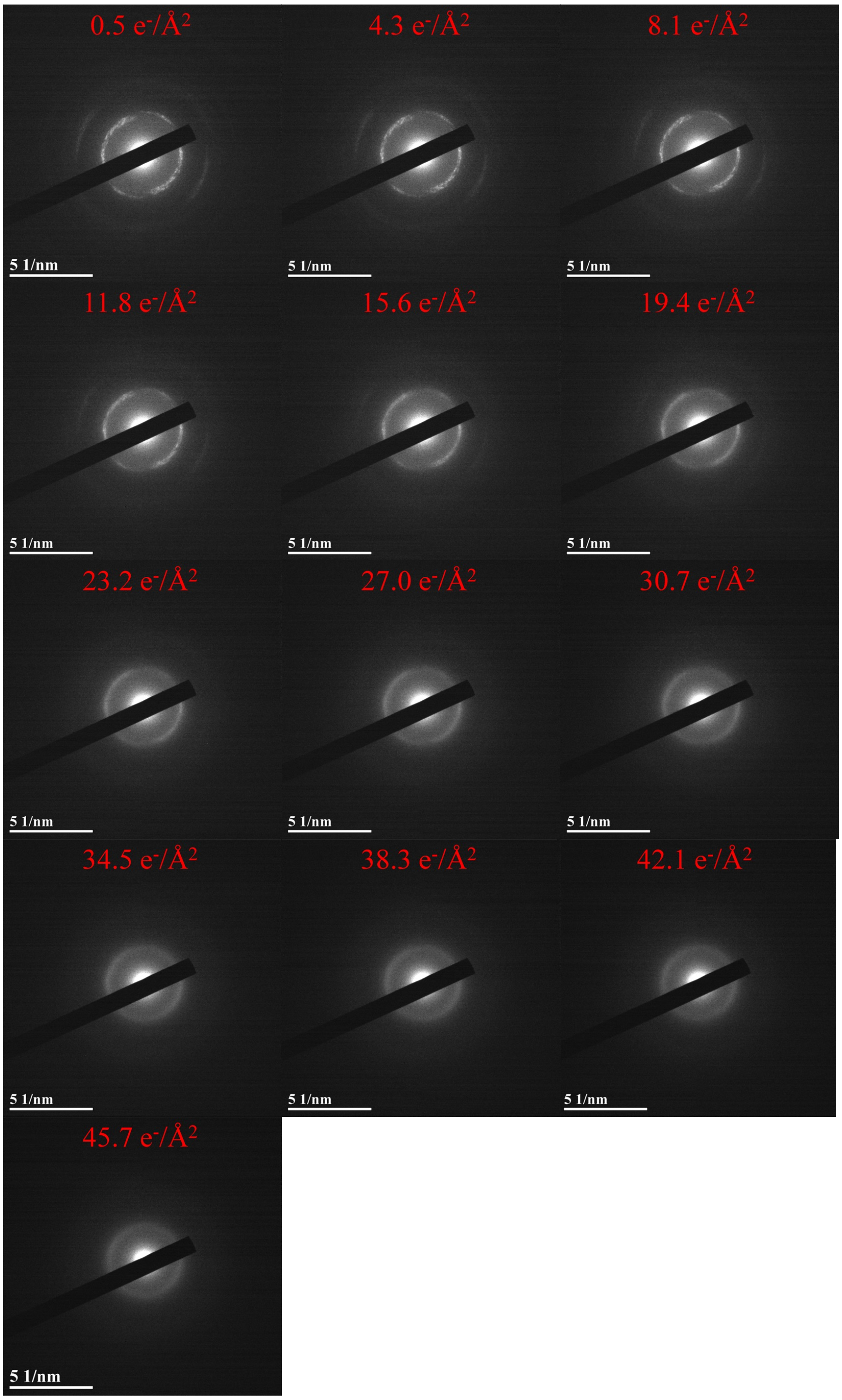

Figure S4. Representative ED patterns from the PE spherulites taken at increasing doses of irradiation. Dose rate ~1.0 $e^-/Å^2·sec$.

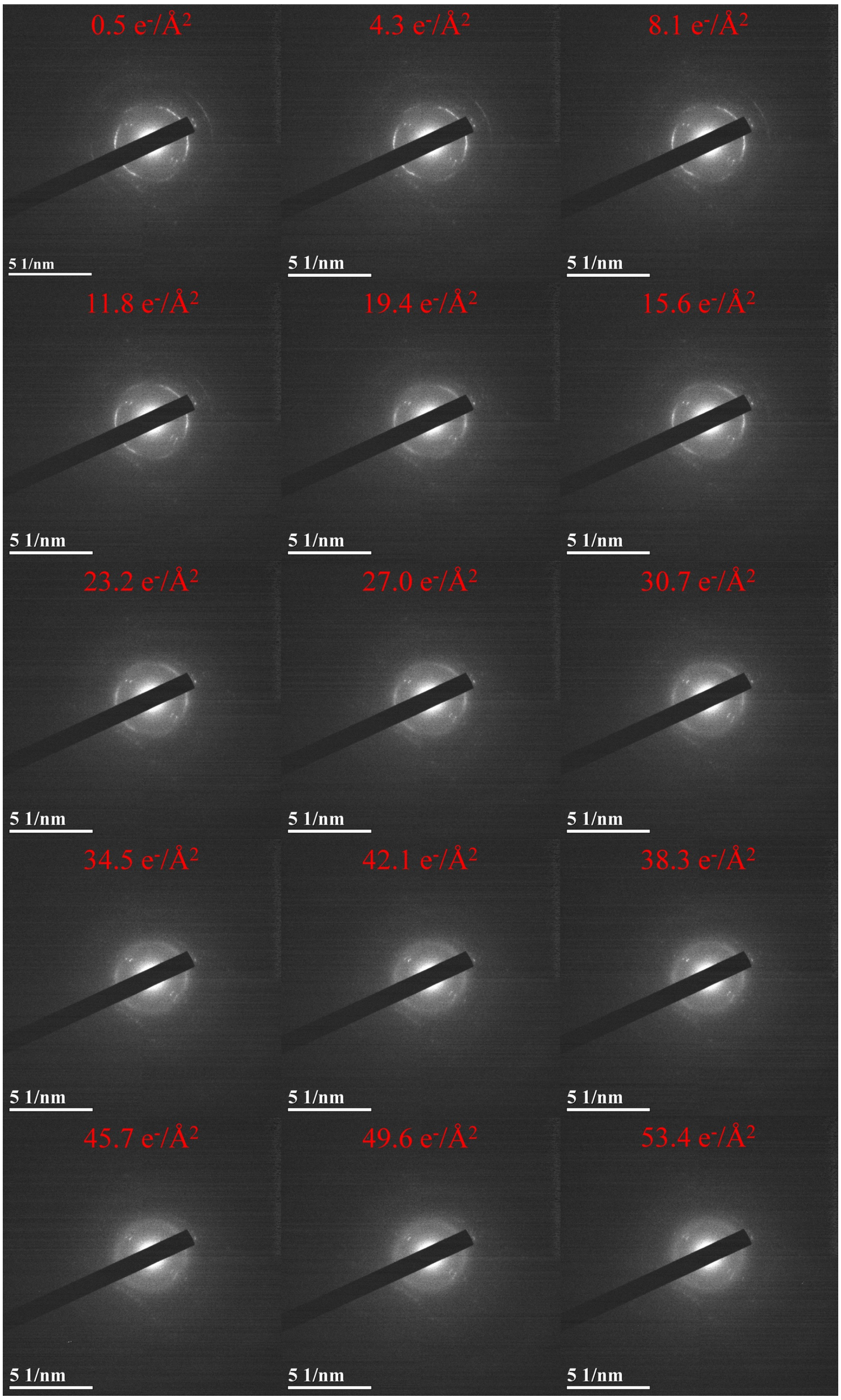

Figure S5. Representative ED patterns from the PE + GA taken at increasing doses of irradiation. Dose rate ~1.0 $e^-/Å^2 \cdot sec$.

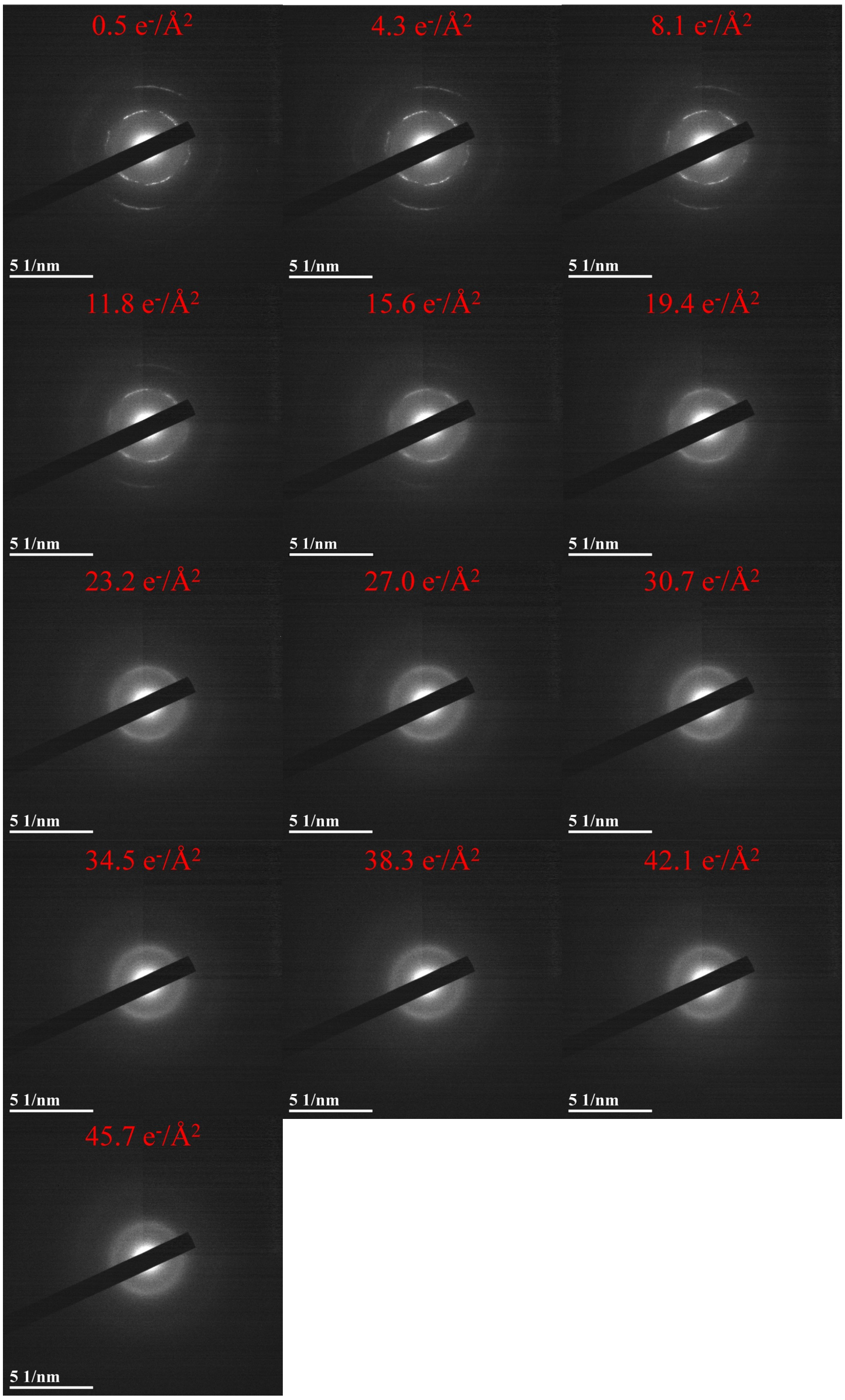

Figure S6. Representative ED patterns from the PE + AA taken at increasing doses of irradiation. Dose rate ~1.0 $e^-/Å^2 \cdot sec$.

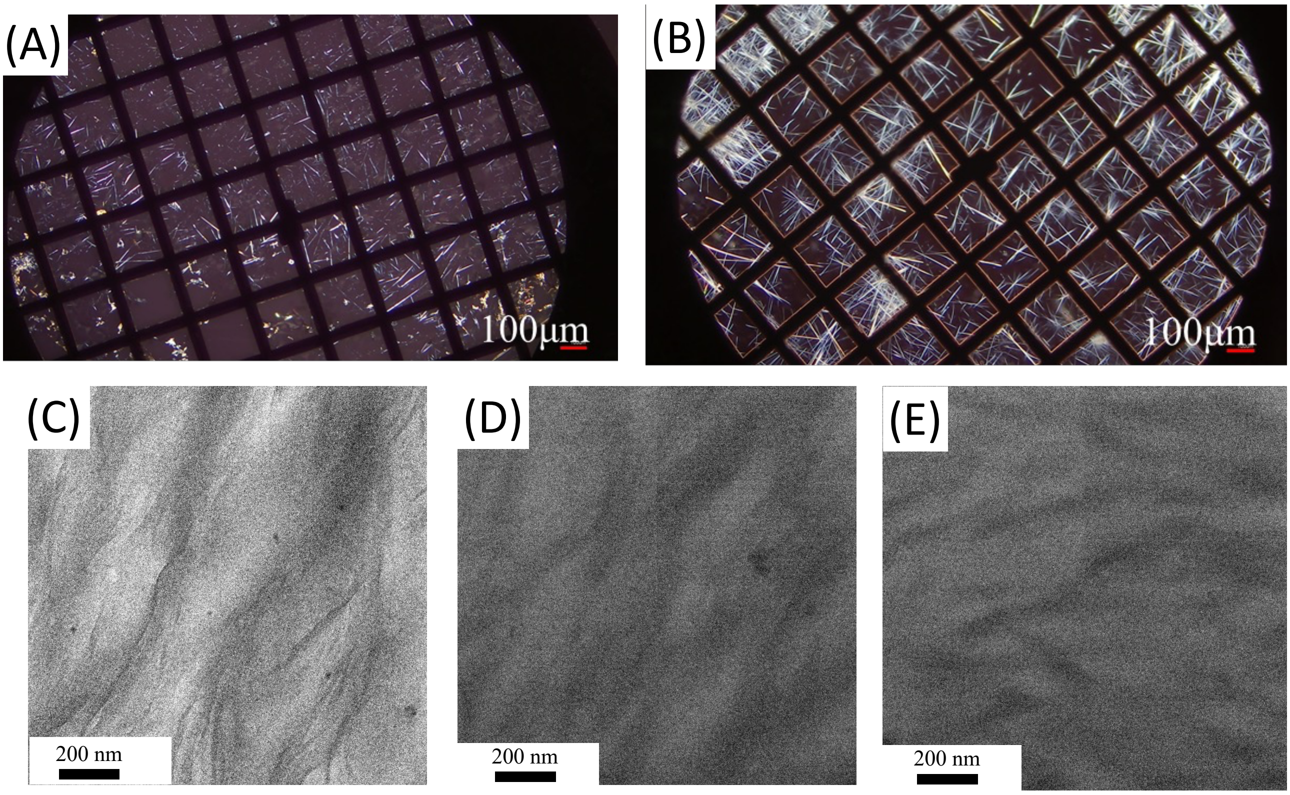

Figure S7. Polarized optical microscopy images and TEM micrographs of PE + GA at (A, C) low (1.6 wt%) and (B, D, E) high concentrations (10 wt%).

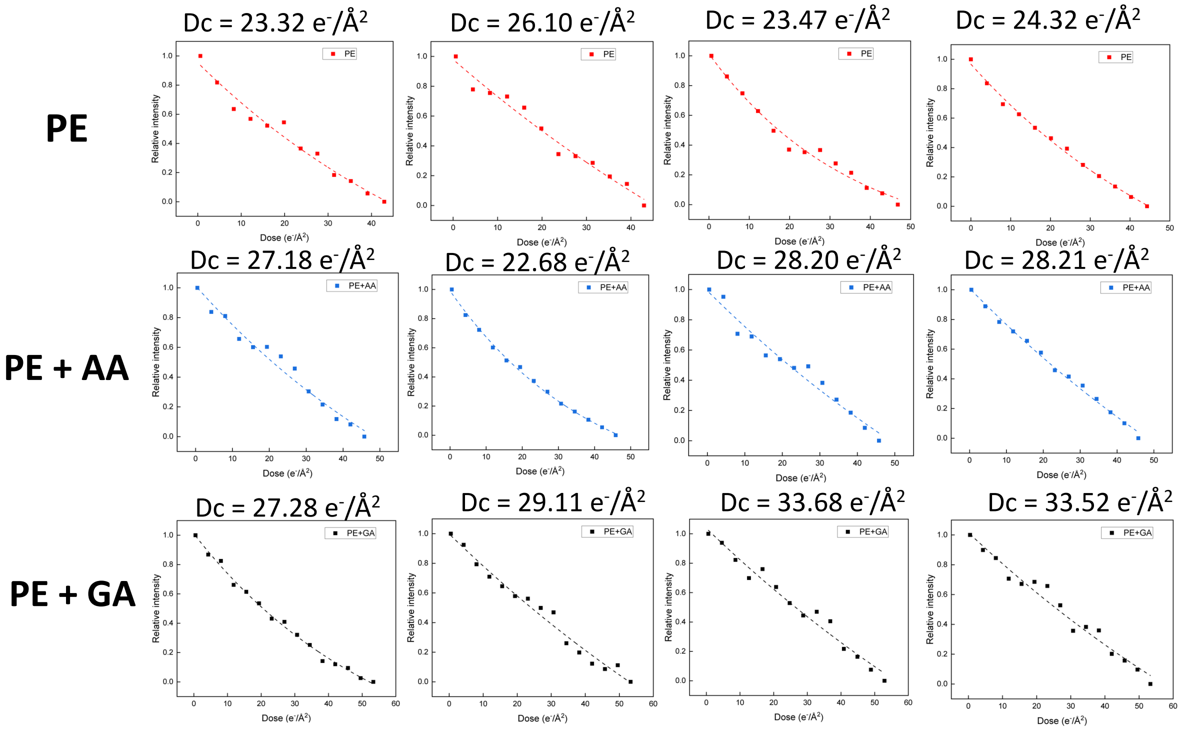

Figure S8. Other data for the relative intensity of $I_{crystal}$ as a function of accumulated dose for PE, PE + GA, and PE + AA, respectively.
